# Effects of *Lactobacillus salivarius* ssp. *salicinius* SA-03 Supplementation on Reversing Phthalate-Induced Asthma in Mice

**DOI:** 10.3390/nu16081160

**Published:** 2024-04-13

**Authors:** Tien-Jen Lin, Chi-Chang Huang, Mon-Chien Lee, Yen-Peng Lee, Wen-Chung Huang, Hsiao-Li Chuang, I-Jen Wang

**Affiliations:** 1 Department of Anaesthesiology, Taipei Medical University-Wan Fang Hospital, Taipei City 116081, Taiwan; trlin1@hotmail.com; 2 Division of Neurosurgery, Department of Surgery, School of Medicine, College of Medicine, Taipei Medical University, Taipei City 110301, Taiwan; 3 Graduate Institute of Sports Science, National Taiwan Sport University, Taoyuan City 333325, Taiwan; john5523@ntsu.edu.tw (C.-C.H.); 1061304@ntsu.edu.tw (M.-C.L.); 4 Graduate Institute of Veterinary Pathobiology, College of Veterinary Medicine, National Chung Hsing University, Taichung City 402202, Taiwan; zoosupreme@gmail.com; 5 Graduate Institute of Health Industry Technology, Chang Gung University of Science and Technology, Taoyuan 333324, Taiwan; wchuang@mail.cgust.edu.tw; 6 National Laboratory Animal Center, National Applied Research Laboratories Research Institute, Taipei 115202, Taiwan; p650214@nlac.narl.org.tw; 7 Department of Pediatrics, Taipei Hospital, Ministry of Health and Welfare, New Taipei City 242033, Taiwan; 8 School of Medicine, National Yang Ming Chiao Tung University, Taipei 112304, Taiwan; 9 College of Public Health, China Medical University, Taichung 400439, Taiwan; 10 National Institutes of Environmental Health Sciences, National Health Research Institutes, Miaoli 350401, Taiwan

**Keywords:** DEHP, probiotics, asthma, *Lactobacillus salivarius*

## Abstract

Probiotics may protect against asthma. We want to investigate whether probiotics can reverse the adverse effects of phthalate exposure on asthma. We selected the female offspring of BALB/c mice, born from pregnant female mice fed with diethylhexyl phthalate (DEHP). They were continuously administrated DEHP and *Lactobacillus salivarius* ssp. *salicinius* SA-03 when they were 5 weeks old, and ovalbumin (OVA) for asthma induction started at 6 weeks for 32 days. The mice were divided into four groups (*n* = 6/group): 1. control group (C), 2. OVA/DEHP group (OD), 3. OVA/DEHP/probiotics low-dose group (ODP-1X), and OVA/DEHP/probiotics high-dose group (ODP-5X). We found that the administration of probiotics significantly reduced the asthma severity of the mice, as well as serum IgE and IL-5. In the ODP-5X group, the proportion of CD4+ cells in the lung was reduced, whereas IL-10 in serum and CD8+ cells in BALF were increased. In histopathology, the ODP group showed reduced infiltration of inflammatory cells, bronchial epithelial cell hyperplasia, and tracheal mucus secretion. These results might indicate that high-dose probiotics may affect anti-inflammatory cytokines and reduce asthma-relative indicators. The above results may provide evidence that high-dose probiotics supplementation might play a modulating role in DEHP causes of allergic asthma in the pediatric animal model.

## 1. Introduction

In 2011, the Taiwan Food and Drug Administration (TFDA) inadvertently discovered that manufacturers had replaced expensive natural emulsifiers in food products with diethylhexyl phthalate (DEHP) for decades [1]. Epidemiological studies have found that DEHP is positively associated with asthma and atopic dermatitis [2,3,4,5]. Maternal exposure to DEHP may lead to an increased incidence of allergic diseases in offspring [6]. Previous studies had found that OVA-induced asthmatic infant Wistar rats experienced aggravated eosinophil and lung inflammation after exposure to DEHP during pregnancy and the lactation period. Moreover, when they were exposed to di(2-ethylhexyl) maleate (DEHM) during pregnancy and lactation, the level of Th2 cytokines was elevated [7].

The improvement of asthma by supplementing probiotics seems to be gaining more and more attention [8]. Previous research indicated that *Lactobacillus rhamnosus* GR-1 administered four times a week for 6 weeks was effective in preventing the elevation of airway total cell counts, lymphocyte counts, and lung IL-1β levels [9]. Another study used different doses of *Lactobacillus plantarum* CQPC11 (LP-CQPC11) to treat ovalbumin (OVA)-induced asthma in BALB/c mice. The results showed that administration of LP-CQPC11 reduced serum levels of OVA-specific IgE, IgE, and OVA-specific IgG1. Furthermore, LP-CQPC11 reduces the activation of the NF-κB pathway and modulates the levels of inflammatory cytokines (TNF-α, IL-4, IL-13, IL-5, and IL-6) in the BALF of asthmatic mice [10]. Recent evidence has demonstrated that probiotics may improve innate immunity disorders by host immune system regulation, including asthma and atopic dermatitis [8,11]. 

*Lactobacillus salivarius* is a probiotic bacterium found mainly in the colon, small intestine, and vagina, but its presence has also been observed in the oral cavity [12]. In colonized areas, it competes with pathogens for a food source and a site of adhesion, thus assisting other friendly strains in the fight against pathogens [13]. By adhering to the intestinal mucosa, it supports the barrier function of the gut. It also exhibits important immunomodulatory effects. In asthmatics, it improves the balance between Th1 and Th2, thus showing anti-inflammatory effects, inhibiting the secretion of pro-inflammatory cytokines and supporting the treatment of numerous skin allergies [14]. By producing lactic acid, bacteriocins, and small amounts of hydrogen peroxide, it inhibits the growth or reduces the number of Candida, *Escherichia coli*, *Salmonella* ssp., *Streptococcus pyogenes*, and *Helicobacter pylori.* Together with *Bifidobacterium breve*, it supports the removal of *Streptococci mutans* (responsible for the formation of tooth decay, plaque, and oral ulceration) in the oral cavity. Promising results were also observed in reducing pain sensation [15]. This is due to the fact that *Lactobacillus salivarius* has the ability to induce the expression of receptors associated with the inhibition of pain responses, particularly OPRM1 in the gut [16]. In addition, it affects the brain–gut connection: by improving gut conditions resulting from antibiotic use or pathogen effects, it can indirectly improve cognitive function and reduce depressive states and anxiety [17]. A positive effect has also been noted on the cardiovascular system, and this is due to a reduction in cholesterol levels and blood pressure [18].

However, little is known about the effect of probiotics on allergic diseases caused by environmental hazards. In this study, we aimed to evaluate whether *Lactobacillus salivarius* ssp. *salicinius* SA-03 supplementation could reverse the adverse effects of phthalate exposure on asthma-by-asthma animal models and to search for the possible mechanism. 

## 2. Materials and Methods

### 2.1. Animals

Pregnant BALB/c mice were selected for the experiment, which was purchased from BioLASCO Taiwan Co., Ltd. (Yilan, Taiwan), and asthma was induced in their female offspring. The mice were provided with Chow 5001 and water *ad libitum* in the animal room of the National Taiwan Sport University (NTSU). The room temperature and humidity were controlled at 24 ± 2 °C and 55 ± 15% under a 12/12 light/dark cycle, respectively. All of the animal experiments in this study were carried out after approval by the Institutional Animal Care and Use Committee (IACUC) of the NTSU, and the study conformed to the guidelines of the IACUC-10721 protocol approved by the IACUC ethics committee.

### 2.2. Measurement of Animal Body Weight and Dietary Intake

The body weight of each group was measured once a week, and the amount of food intake was weighed by subtracting the amount of remaining food from the food added during the study. 

### 2.3. Establishment of the DEHP Causes of Allergic Asthma in the Pediatric Animal Model

The animal model was prepared as per the following method: Pregnant BALB/c mice were selected for olive oil intervention during pregnancy and lactation, for which the oil was mixed with DEHP (400 μg/kg) [19]. The asthmatic disease was induced in the OD, ODP-1X and ODP-5X group by OVA when the offspring were 6–8 weeks old. The DEHP and probiotics were simultaneously administered until the sacrifice of the animals.

### 2.4. Sample Preparation

The probiotic *Lactobacillus salivarius* ssp. *salicinius* SA-03 used in the study was isolated from the feces of gold medalist Wei-Ling Chen, who won the 2008 Olympic women’s 48 kg weightlifting [20]. The safety of the strain was identified by a third-party independent testing institution (Food Safety Inspection Center of Asia University), with the 16S–23S rRNA test for the *Lactobacillus saliva*. The SA-03 was processed by Bioflag Biotechnology Co., Ltd (Tainan, Taiwan). in a dry powder form. Prior to supplementation, the powder was dissolved in phosphate-buffered saline (PBS) at pH 7.2. The dose was based on the daily human recommended intake of 1 × 10^10^ CFU [21]. According to the U.S. Food and Drug Administration, the equivalent dose (HED) can be calculated by the conversion factor of the mouse species between humans of 12.3, which was based on the human body surface area. Thus, the dose for the mice was 2.05 × 10^9^ CFU/kg body weight; the five-fold dose was 1.03 × 10^10^ CFU/kg body weight.

### 2.5. Asthmatic Animal Model

The female offspring were used in our experiment, born from the pregnant BALB/c mice administered the DEHP. On the 1st and 14th days of the experiment, the OD, ODP-1X, and ODP-5X mice were intraperitoneally injected with 0.1 mL of a 20% OVA solution as an allergen and adjuvant that combined 2.25 mg of Al(OH)_3_ and PBS to induce systemic allergy. On the 28th day, gaseous 1%OVA was administered to the mice for three consecutive days to induce local allergy in the respiratory tract. Airway hyperresponsiveness (AHR) was measured on day 32. For the control group, normal saline was used instead of OVA administration.

### 2.6. Group of Experimental Animals

**Figure 1:**
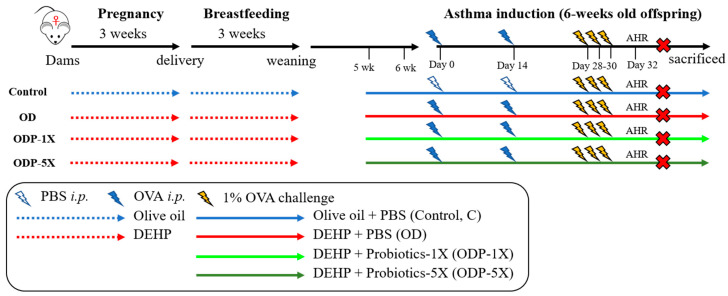
Experimental design for DEHP causes of allergic asthma in the pediatric animal model. Procedure of DEHP exposure and induction of asthma. Dams were exposed to DEHP during pregnancy until weaning when pups were 3 weeks old. *i.p.* = intraperitoneal, AHR = airway hyper-responsiveness.

The experimental animals used the female offspring of pregnant mice given olive oil (control group) or DEHP. When the animals were 5 weeks of age, they were given DEHP and probiotics until the animals were sacrificed. Asthma was induced when the animals were 6 weeks of age. The experimental animals were divided into four groups, each with 6 animals, namely, the control group (C), OVA/DEHP group (OD), OVA/DEHP/probiotics low-dose group (ODP-1X), and OVA/DEHP /probiotics high-dose group (ODP)-5X). SA-03-1X was fed with 2.05 × 10^9^ CFU/kg; SA-03-5X was fed with 1.03 × 10^10^ CFU/kg. All animals were sacrificed the next day after the airway hypersensitivity was measured. The experimental procedure is shown in Figure 1.

### 2.7. Airway Hyper-Responsiveness (AHR)

The airway hyper-responsiveness was measured by methacholine (Sigma-Aldrich, St Louis, MO, USA), which is a non-specific tracheal contracting agent acting on the muscarinic neurotransmitter receptor of the bronchial parasympathetic plexus, such as acetylcholine. If a patient with potential asthma reacts with this agent, their bronchial smooth muscle contracts, and asthma thus occurs. On the 32nd day after OVA induction, the airway hypersensitivity of the mice was measured to observe any respiratory tract contraction. The mice were exposed to concentrations of methacholine at 0 (saline), 12.5, 25, and 50 mg/mL by steam inhalation (spray) for 3 min. The mice were then examined in a chamber equipped with a respiratory hypersensitivity detection system (BUXCO Electronics, Inc., Wilmington, NC, USA) for 3 min. The sensor detected the respiration and airflow variation of the mice, and the data were passed to the BioSystem XA software (Buxco Electronics, Troy, NY, USA) for analysis to obtain the Penh value of respiratory hypersensitivity.

### 2.8. Hematology Analysis

The blood of the animals was sampled after sacrifice. The blood was stored in a collection tube containing EDTA anticoagulant and mixed uniformly at room temperature. The white blood cells (WBCs) were analyzed by a complete blood count (CBC) analyzer, and then the neutrophil, lymphocyte, mononuclear cell, eosinophil, and basophil percentages were analyzed. The eosinophils were counted as one of the indicators for assessing the severity of asthma.

### 2.9. Serum OVA-Specific IgE and IgG1 Measurements

A 96-well ELISA plate (TPP, Trasadingen, Switzerland) was coated with 10 μg/mL of OVA (dissolved in 1X PBS) at 4 °C overnight. Then, blocking proceeded with 3% bovine serum albumin (BSA) (in PBS) at 37 °C. After 1 h, the sample was added and allowed to stand for 1 h. Biotinylated rat anti-mouse monoclonal IgE or IgG1 (BD Biosciences, San Jose, CA, USA) was added for another 1 h at 37 °C for reaction. Then, streptavidin conjugated-HRP was added and reacted for 30 min at room temperature. A dye coupler was added and reacted for 20 min at room temperature—a solution that contains 2 mg of o-phenylenediamine dihydrochloride (OPD; Sigma-Aldrich) and 2 μL of 30% H_2_O_2_ dissolved in 5 mL of 1X phosphate citrate buffer. Finally, the reaction was stopped with 25 μL of 3 M H_2_SO_4_. The absorbance was measured with an ELISA reader (Tecan, Mannedorf, Switzerland) at a wavelength of 450 nm.

### 2.10. Collection of Bronchoalveolar Lavage Fluid (BALF)

The sacrifice was performed with 1 mL of Zoletil 50. Approximately 0.5 mL of 1 mL BALF was collected via three extractions from the lung, a procedure that involved injecting saline into the lungs with a 24 G residual hose needle and a 1 mL syringe connected to a PE60 hose.

### 2.11. Measurement of Cytokines

The concentrations of the IL-4, IL-5, IL-10, IL-13, and INF-γ cytokines in alveolar lavage fluid and serum were measured by a Luminex analyzer. The corresponding antibodies were used to coat microbeads using a Milliplex kit (Millipore, Billerica, MA, USA) based on the immunological antibody–antigen bond. The beads inside the Milliplex kit consisted of infrared and far-infrared fluorescent dyes in different proportions to form 100 color codes. Coded beads can be attached to antibodies with specific proteins to identify the protein in a sample. A detection antibody specific to biotin was added and reacted. Finally, streptavidin phycoerythrin (SAPE) was added as the fluorescent antibody reaction. Milliplex can be used for qualitative analysis and to quantify multiple substances in a well.

### 2.12. Eosinophil Smear

First, 10 μL of cell suspension was dropped onto a glass slide for Liu’s staining. For this, Liu’s stain A was dripped onto the glass slide for 30 s, then the B agent was dripped for 4 min. The back of the slide was rinsed with tap water for 20 s and then dried. The dried slides were mounted with a 24 × 40 mm coverslip. When the gel was wholly air-dried, the cells were observed under an optical microscope with oil immersion. The number of eosinophils was calculated in 200 cells to estimate the total number of eosinophils in 1 mL of lung wash.

### 2.13. Flow Cytometry Analysis

The red blood cells in BALF were removed by ACK lysis buffer (Becton-Dickinson Biosciences, Ann Arbor, MI, USA), then the BALF cells were suspended in FACS buffer (Becton-Dickinson Biosciences) for staining with monoclonal antibodies 9cd4, cd8, cd3, cd19, and foxp3. Finally, these were detected by an Accuri^TM^ C6 flow cytometer (Becton-Dickinson Biosciences) and analyzed using BD Accuri^TM^ C6 software (BD Biosciences, San Jose, CA, USA).

### 2.14. Histopathological Evaluation

OVA was used to induce chronic lung lesions in the mice. All single leaves of the left lung were sampled to generate a transverse section in the middle. The tailored tissues were stained with H&E and Periodic acid–Schiff (PAS) for observation. The evaluation was conducted with six samples per group. The evaluation method was slightly modified from the methodology of a previous study [22]. Three different histological changes were quantified for the histopathological evaluation: bronchial epithelial hyperplasia (severity score: 0–4 points), lung inflammation (severity score: 0–4 points), and bronchial mucus secretion (PAS staining) (severity score: 0–4 points). The severity was graded on a scale of 0–4, with 0 referring to no response and grade 4 representing the most severe response. Finally, the three scores were summed up as a comprehensive score index for asthma.

### 2.15. Statistical Analysis

All data were analyzed by one-way ANOVA using the Prism computer statistical software package. Tukey’s test was used for the post-hoc test. There is a significant difference between the groups when *p* < 0.05. All data are presented as mean ± SEM.

## 3. Results

### 3.1. Body Weight and Dietary Intake

**Figure 2:**
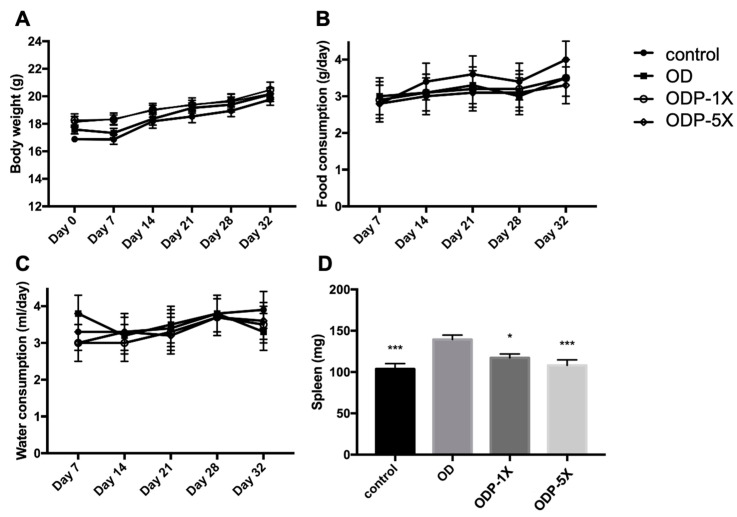
The effects of *Lactobacillus salivarius* ssp. *salicinius* SA-03 on the DEHP causes of allergic asthma in the pediatric animal model. Comparison of the (**A**) body weight changes, (**B**) food consumption, (**C**) water consumption, and (**D**) spleen weight of each group. Data are presented as mean ± SEM; * *p* < 0.05 and *** *p* < 0.005 when compared to the OD group.

The average body weight of the mice during the study is shown in Figure 2A. There was no significant difference in body weight among the groups (*p* > 0.05). The average food and water intake of the mice is shown in Figure 2B, C. No significant difference was observed in the food and water consumption between the dose and control groups (*p* > 0.05). The spleens of the mice were weighed after sacrifice, as shown in Figure 2D. The spleen weight of the OD group was significantly higher, whereas that of the probiotics group was significantly lower (*p* < 0.05).

### 3.2. Lactobacillus salivarius ssp. salicinius SA-03 Reduces the Airway Hypersensitivity Caused by OVA/DEHP

**Figure 3:**
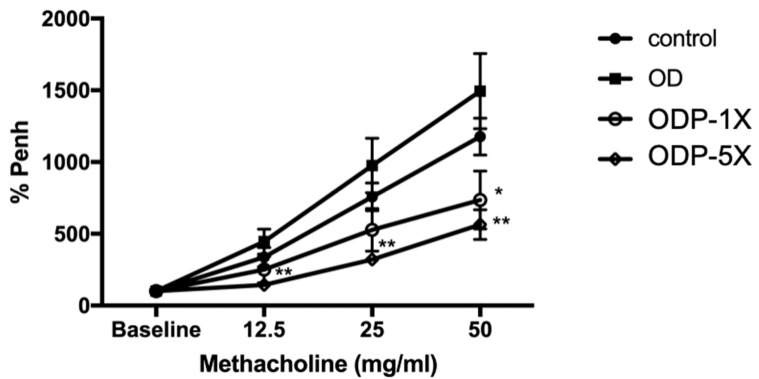
Effect of *Lactobacillus salivarius* ssp. *salicinius* SA-03 on airway hyper-responsiveness (AHR) in mice. The airway responsiveness (Penh%) of the probiotics (SA-03)-treated groups was significantly decreased compared with the OD model group. Data are presented as mean ± SEM; * *p* < 0.05, ** *p* < 0.01 when compared to the OD group.

The results of the airway hypersensitivity measurement after the 32-day asthma induction of the offspring are shown in Figure 3. The OD group had the greatest increase in the Penh% respiratory hypersensitivity coefficient (the ratio with baseline). There were significant differences between the OD and control groups in the methacholine test with different concentrations (12.5 mg/mL: 4.46 times vs. 1.44 times; 25 mg/mL: 9.76 times vs. 3.21 times; 50 mg/mL: 14.95 times vs. 5.64 times; *p* < 0.01). The performance of the ODP-5X group was significantly lower than that of the OD group in the 50 mg/mL methacholine test. For the 50 mg/mL methacholine test, the respiratory hypersensitivity coefficient of the OD group was the highest among the four groups, and the respiratory hypersensitivity coefficient of the ODP-5X group was lower than that of the control group by 2.03 and 2.65 times.

### 3.3. Lactobacillus salivarius ssp. salicinius SA-03 Reduces the OVA-Specific IgE Level in Serum and OVA-Specific IgG1 Level in Lung Lavage Fluid

**Figure 4:**
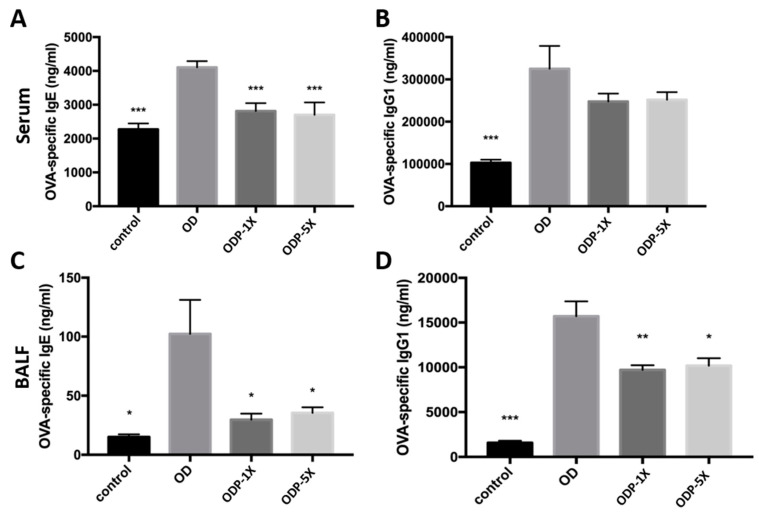
*Lactobacillus salivarius* ssp. *salicinius* SA-03 inhibits serum (**A**) IgE, (**B**) IgG1 and bronchoalveolar lavage fluid (BALF) (**C**) IgE, (**D**) IgG1 immunoglobulin levels. Concentrations of OVA-specific IgE and IgG1 immunoglobulin in the serum and BALF. Data are presented as mean ± SEM; * *p* < 0.05, ** *p* < 0.01, and *** *p* < 0.005 when compared to the OD group.

After OVA administration, OVA-specific IgE and IgG1 immunoglobulin associated with asthma were produced. As shown in Figure 4A, the OVA-specific IgE serum concentration of the OD group was 4106.5 ± 183.8 ng/mL, which was significantly greater than that of the other groups. The OVA-specific IgE serum concentrations of the ODP-1X and ODP-5X groups were significantly lower than that of the OD group (*p* < 0.005). However, there was no significant difference between the ODP-1X and ODP-5X groups (ODP-1X: 2812.7 ± 233.3 ng/mL; ODP-5X: 2700.5 ± 368.2 ng/mL). The OD group had the highest OVA-specific IgE concentration in their lung lavage fluid, whereas that of the ODP-1X and ODP-5X groups was significantly lower (*p* < 0.05) (Figure 4C). The OVA-specific IgG1 serum concentration of the control group was significantly lower than that of the OD group (control: 102,936 ± 7121 ng/mL; OD: 325,194 ± 53,859 ng/mL) (Figure 4B). For the lung lavage fluid, the concentration of OVA-specific IgG1 of the OD group was significantly greater than that of the control group (OD: 157,09 ± 1668.2 ng/mL; control: 1566.7 ± 238.8 ng/mL) (*p* < 0.005). The concentrations of OVA-specific IgG1 of the ODP-1X and ODP-5X groups (1X and 5X) were significantly lower than that of the OD group (ODP-1X: 9714.5 ± 504 ng/mL, *p* < 0.01; ODP-5X: 10,167 ± 840 ng/mL, *p* < 0.05) (Figure 4D).

### 3.4. Effects of DEHP and Lactobacillus salivarius ssp. salicinius SA-03 on Asthma-Related Cytokines in Serum and BALF

**Figure 5:**
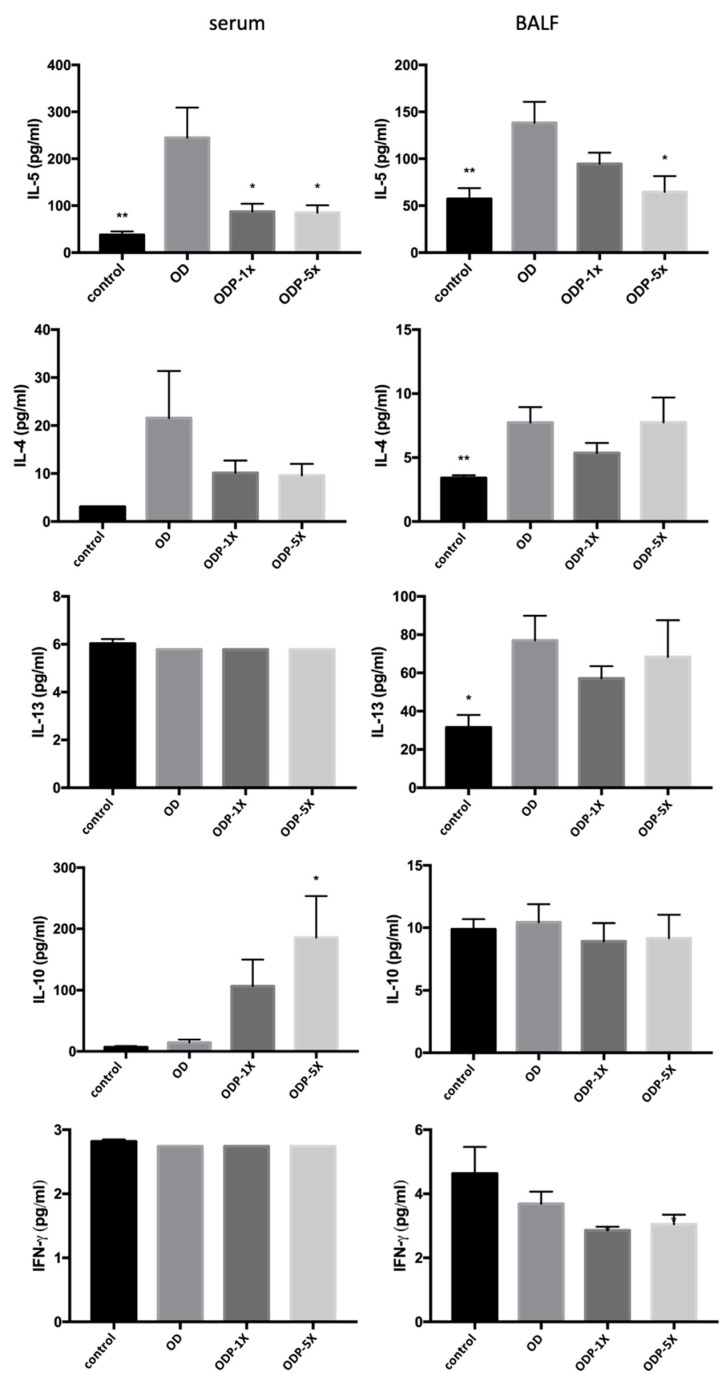
Level of Th2 cytokines in the serum and bronchoalveolar lavage fluid (BALF). Data are presented as mean ± SEM; * *p* < 0.05 and ** *p* < 0.01 when compared to the OD group.

The results are shown in Figure 5. The OD group showed a significant increase in IL-5 concentration in the blood and bronchoalveolar lavage fluid (BALF). The serum concentration of IL-5 of the OD group was significantly greater than that of the control group, whereas the concentrations of the ODP-1X and ODP-5X groups were significantly lower than that of the OD group (*p* < 0.05). For the BALF, the IL-5 concentrations of the control and ODP-5X groups were significantly lower than that of the OD group (control: *p* < 0.01; ODP-5X: *p* < 0.05). On the contrary, the ODP-5X group’s serum concentration of IL-10, an interleukin that regulates Th1–Th2 balance, was significantly greater than that of the OD group (*p* < 0.05). No significant difference was observed in the concentrations of BALF IL-10 and serum IL-4, IL-13, and INF-γ cytokines among the groups. The OD group’s BALF concentration of IL-13, an interleukin that promotes IgE production by B cells, was significantly greater than that of the control group, whereas there was no significant difference between the ODP-1X, ODP-5X, and OD groups. Collectively, DEHP combined with OVA can promote the activation of IL-5 cytokines in the blood and adjacent bronchial tissues in the lung, and intervention with probiotics can inhibit an increase in IL-5.

### 3.5. Effects of DEHP and Lactobacillus salivarius ssp. salicinius SA-03 on Asthma-Related Eosinophils in Blood and BALF

**Figure 6:**
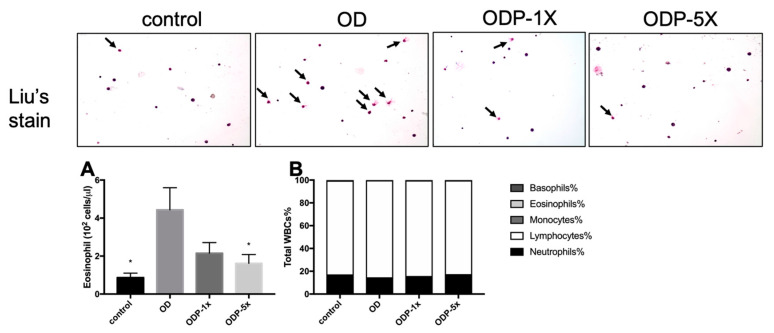
Content of (**A**) eosinophils in bronchoalveolar lavage fluid (BALF) and (**B**) whole blood white blood cell analysis. The BALF was made into a smear for Liu’s staining and observed under a 200-fold field of view; the blood was detected by a fully automated blood cell counter. The arrows show eosinophilic granulocyte. Data are presented as mean ± SEM; * *p* < 0.05 when compared to the OD group.

The bronchoalveolar lavage fluid (BALF) was extracted after sacrifice. Then, 10 μL of the cell suspension was dropped onto a glass slide for Liu’s staining, after which the number of eosinophils was observed under an optical microscope. Based on the results, the number of eosinophils in the ODP-5X group was significantly reduced (*p* < 0.05). As shown in Figure 6A, the collected blood was stored in a collection tube containing EDTA anticoagulant, and the white blood cells (WBCs) were classified by an automatic blood analyzer. There was no significant difference in the mononuclear spheres, eosinophils, and basophils between the groups (Figure 6B).

### 3.6. Effects of Plasticizers and Lactobacillus salivarius ssp. salicinius SA-03 on Various Immune Cells

**Figure 7:**
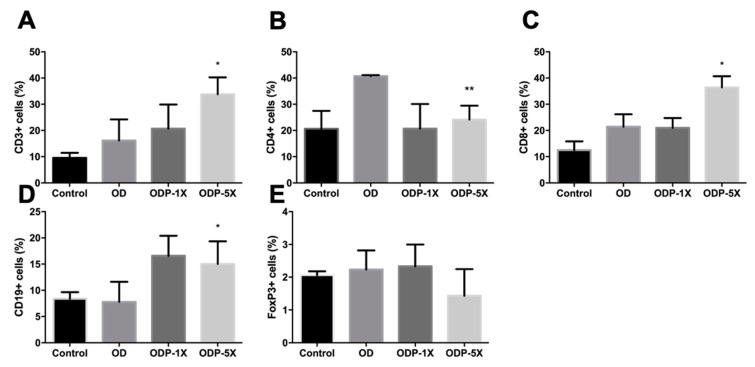
The effect of *Lactobacillus salivarius* ssp. *salicinius* SA-03 on the percentage of immune cells in BALF. Using flow cytometry to analyze the percentage of immune cells in BALF of (**A**) CD3, (**B**) CD4, (**C**) CD8, (**D**) CD19, and (**E**) Foxp3 among the groups. Data are presented as mean ± SEM; * *p* < 0.05 and ** *p* < 0.01 when compared with to OD group.

As shown in Figure 7, the monoclonal antibodies were specific to CD4 for helper T cells, CD8 for cytotoxic T cells, CD3 for total T cells, FOXP3 for regulatory T cells, and CD19 for B cells. It is generally believed that an increase in CD4+ cells promotes asthma-related cytokines, resulting in bronchial inflammation and other symptoms, but CD8+ cells act oppositely. Regulatory T cells can reduce type 2 killer T cells to alleviate bronchial inflammation and asthma. B cells can promote immunoglobulin secretion, which interferes with the degranulation of mast cells and leads to respiratory tract overreaction caused by increased inflammatory mediators. Both T cells (CD3+) and B cells (CD19+) are part of the lymphatic system. For the BALF, the ratio of T cells (CD3+) and B cells (CD19+) in the ODP-5X group was significantly greater than that in the OD group (*p* < 0.05). The proportion of T helper cells (CD4+) in the T cells of the ODP-5X group was significantly lower than that of the OD group (*p* < 0.01). The proportion of T sputum cells (CD8+) in the ODP-5X group was significantly greater than that in the OD group (*p* < 0.05). However, there was no significant difference in regulatory T cells (FOXP3) between the groups.

### 3.7. Lactobacillus salivarius ssp. salicinius SA-03 Improves OVA/DEHP-Induced Lung Tissue Lesions

**Figure 8:**
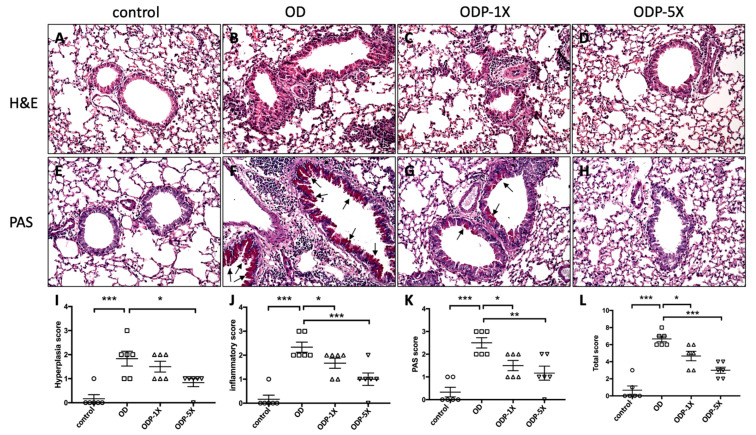
*Lactobacillus salivarius* ssp. *salicinius* SA-03 suppress OVA-induced airway hyperresponsiveness, lung inflammation, and mucus production. The lung histopathology was examined for the presence of epithelial (**I**) hyperplasia, (**J**) inflammatory response, (**K**) mucus secretion and (**L**) total score. (**A**–**H**) Each section was subjected to H&E and PAS staining and observed under a 200-fold field of view. The arrows indicate areas of increased mucus secretion. Data are presented as mean ± SEM; * *p* < 0.05, ** *p* < 0.01, and *** *p* < 0.005 when compared to the OD group.

On morphological observation, the OD group showed obvious epithelial cell hyperplasia and hypertrophy in the lung bronchi. Bronchial thickening and perivascular inflammatory cell infiltration were observed, as shown in Figure 8B. The mucus secretion of endocervical epithelial cells was evaluated by PAS staining. The amount of mucus in the bronchial epithelial tissue of the OD group was significantly increased, as shown in Figure 8F, and the arrows indicate the mucus secretion. For the ODP-1X and ODP-5X groups, the epithelial cell proliferation, inflammatory reaction, and mucus secretion in the lung tissues were improved when compared to the OD group. There were significant differences among the groups when the results were quantified (*p* < 0.05; OD group vs. ODP-1X group and OD group vs. ODP-5X group) (Figure 8I–L).

## 4. Discussion

This is one of the first few studies to evaluate the protective effect of probiotics supplementation against the endocrine disruptor DEHP derived from the background environment. Animal allergy models must reproduce the process and reactions observed in humans as closely as possible. Hence, in our mouse model of asthma, major indicators relating to the asthma model (e.g., airway hypersensitivity, total serum IgE, IFN-γ, IL-4, IL-10, IL-13, and inflammatory cell count in BALF) were detected to evaluate the physiological and biochemical changes induced by the antigen (OVA). Among these biomarkers and indicators associated with our mouse model of asthma, airway hypersensitivity is of extreme importance. Our results showed that the spleen weight of the OD group was significantly heavier, whereas that of the probiotics group was significantly reduced (*p* < 0.05). This result indicated that the DEHP/OVA-induced asthmatic model was successfully established, and probiotics may play a certain role in modulating the immunopathological processes taken in developing asthma. Thickening of the sub-basement membrane, disproportionate secretion of mucus, subepithelial fibrosis, inflammatory cell infiltration, and extracellular matrix deposition in the subepithelial layer can contribute to airway structural changes in responding to DEHP/OVA induction [23]. In our study, high-dose probiotics inhibited the infiltration of inflammatory cells in the lungs and reduced bronchial epithelial cell hyperplasia and tracheal mucus secretion, thus decreasing airway hypersensitivity. The total IgE levels in serum showed that a promoted effect evoked by DEHP exposure contributed to the development of OVA-induced asthma or asthma-like symptoms. Furthermore, the concentration of OVA-specific immunoglobulin IgE in serum or BALF was significantly reduced in the probiotic intervention group, indicating that probiotics may have an inhibitory effect on DEHP/OVA-induced OVA-specific immunoglobulin IgE. For the serum concentration of IgG1, the probiotic intervention group showed a significant decrease when compared to the OD group only in BALF, indicating that the probiotics had a systematic inhibitory effect on DEHP/OVA-induced OVA-specific immunoglobulin IgG1.

On the panel of cytokine regulation, patients with allergic respiratory diseases show a trend toward an increase in the Th2 cell population [24]. The IL-4, IL-5, and IL-13 secreted by Th2 inhibit the activity of Th1 and stimulate B cells to produce IgG1 and IgE, and their secretion regulates the allergy-mediated Th1–Th2 balance, lymphocyte infiltration, and cytokine secretion. IL-5 has a key role in the migration, maturation, and survival of eosinophils. Meanwhile, studies have shown that Th2-mediated eosinophilic leukemia occurs when there is exposure to allergens. The Th1 cytokines IFN-γ and IL-10 act as negative feedback to Th2 cells to reduce specific-IgE production [25,26]. For asthma-associated cytokine secretion, IL-5 is primarily a T cell-derived cytokine, which is particularly important for the activation and survival of eosinophils [27,28]. In our study, the proportion of CD4 in the helper T cells of the OD group significantly increased, and the serum and BALF IL-5 concentrations increased by stimulation with DEHP/OVA but decreased in the probiotic intervention group. This is consistent with the results of BALF CD4 and eosinophils, indicating that IL-5 is associated with increased local CD4 T cells and eosinophilic globules. Moreover, studies have confirmed that IL-5 can promote eosinophilic activity and cause an increase in IgE [29]. Therefore, we speculate that the increased IgE in the OD group was mainly caused by IL-5 to stimulate eosinophils. On the contrary, the increased serum IL-10 concentration may be associated with an increased CD8 T cell population. Many studies have confirmed that IL-10 has anti-inflammatory benefits that alleviate the symptoms of asthma because of the increased CD8 T cell population and the reduced conversion of IgE [30,31]. IL-10 inhibits eosinophils and has the ability to generate IgE [32,33]. A similar response was observed in our study in the high-dose probiotics group. In recent years, supplementation with probiotics has been proven to be one of the most effective ways to regulate gut microbiota to improve the respiratory system and asthma-related diseases. In addition, recent clinical trials and animal experiments have shown that probiotics may inhibit the progress of allergic responses. For instance, the *E. coli* strain Nissle 1917 (EcN) alleviates the asthmatic pattern induced by mouse ovalbumin (OVA), and a mechanism behind this may be the decreased IL-5 level, increased IFN-γ-generated type 1 T helper cells, and more IFN-γ produced in the trachea [34]. In a DSS-induced enteritis model, EcN has been shown to improve enteritis via the regulation of inflammatory-related micro-RNA (miR-143, miR-150, miR-155, miR-223, and miR-375) expression [35]. Moreover, *Lactobacillus fermentum* L930BB (L930BB) and *Bifidobacterium* subsp. *imida* IM386 (IM386) have been demonstrated to slow the severity of enteritis by an anti-apoptotic pathway involved in PI3K/Akt [19]. A previous study used BALB/c mice to sensitize the asthma model with ovalbumin (OVA) to investigate the response of potential probiotics to antigen challenge. The results showed that real-time oral treatment of *Lactobacillus salivarius* PM-A0006 (10^6^–10^7^ CFU) significantly reduces the influx of eosinophils into the tracheal cavity and reduces the serum OVA specificity in the BALF of antigen-infected animal IgE and the levels of eosinophil chemotactic factor. In addition, PM-A0006 reduces airway hyperresponsiveness caused by allergens and increases interferon (IFN)-γ levels [24]. *L. salivarius* might participate in the pathogenesis of bronchial asthma through CD4 + CD25 + Foxp3 + Treg cells, increase the expression of T-bet mRNA, and inhibit the expression of GATA-3 mRNA at the transcriptional level to improve the Th1–Th2 imbalance. In addition, a previous study confirmed that the level of IFN-γ in the supernatant of the splenocyte culture of the simple gavage group of *L. salivarius* was higher than that of the control group, and the level of IL-4 was lower [14]. In the mixed strain test, the use of *L. salivarius* LS01 and *Bifidobacterium breve* BR03 to supplement probiotics at a ratio of 1:1 effectively decreases the secretion of proinflammatory cytokines by PBMCs, leading to an intense increase in IL-10 production, aiding the maintenance of the physiological profile of the immune response in mucosal lymphoid tissue, and this has also been shown to have immunomodulatory effects [36]. In the current study, we used SA-03 to confirm this mechanism and efficacy.

It was found that among the mother–child lifestyle and environmental factors for the risk of neonatal sensitization, the metabolites of butyl benzyl phthalate (BBP) directly affect the severity of asthma in offspring, which continues to the F2 generation [37]. These results provide strong evidence that maternal exposure to BBP alters the expression of the genes involved in Th2 differentiation through epigenetic changes, increasing the risk of allergic asthma in offspring. The airway microbiota in early life might interact with the development of the immune system and lead to the development of childhood asthma [38]. For children with early allergic allergies, the colonization of *Haemophilus*, *Streptococcus*, and *Moraxella* in the upper respiratory tract increases the risk of chronic wheezing at five years old. The allergen-specific IgE levels of these children with early allergies have been detected at six months of age [39]. In recent years, as the intestinal–gut axis crosstalk effect has been gradually confirmed, the diversity, richness, and energy utilization system of the intestinal flora have become more and more important for the impact of immunity, inflammation, lungs, and the respiratory tract [40,41]. Compared to non-asthmatic children, school-age children with asthma show lower gut microbiome diversity at one month of age [42]. Compared to healthy controls, the abundance of *Roseburia* (phylum *Firmicutes*) and *Faecalibacterium* are lower, whereas genera *Clostridium* (phylum *Firmicutes*) and *Enterococcus* are higher [43]. A past study pointed out that the abundance of *Lachnospira*, *Veillonella*, *Faecalibacterium*, and *Rothia* in the gut of asthmatic infants is significantly reduced. However, inoculation of these bacteria in germ-free (GF) mice could improve airway inflammation and prevent the development of asthma [44]. The OVA-induced IL-5 cytokine sensitization was suppressed by high doses of probiotics relieving the inflammatory cell infiltration in the lungs; thus, the asthma allergic reaction was suppressed. 

In histopathology, higher-dose probiotics can differentially inhibit the infiltration of inflammatory cells in the lungs and subsequently reduce bronchial epithelial cell hyperplasia and tracheal mucus secretion.

## 5. Conclusions

Our results indicated that when mice are exposed to DEHP during pregnancy and lactation, the OVA-induced lung lesions in the progeny are aggravated, and the OVA-induced IL-5 cytokines in the progeny increase. When the progeny was induced by DEHP OVA as asthma, high doses of *Lactobacillus salivarius* ssp. *salicinius* SA-03 relieved the inflammatory cell infiltration in the lungs, thereby reducing the number of eosinophils, the bronchial epithelial cell proliferation, and the mucus secreted by goblet cells, resulting in an improvement in respiratory allergic reactions.

There is substantial evidence that DEHP results in increased allergies, and that probiotics supplementation may play a modulating role in the adverse effects of DEHP. Further clinical trials may address the impact of probiotics supplementation strategies in the prevention of DEHP-related adverse effects in areas where people are destined to be exposed.

## Data Availability

The original contributions presented in the study are included in the article, further inquiries can be directed to the corresponding author.

## References

[1] Huang C.F., Wang I.J. (2017). Changes in Urinary Phthalate Metabolite Levels before and after the Phthalate Contamination Event and Identification of Exposure Sources in a Cohort of Taiwanese Children. Int. J. Environ. Res. Public Health.

[2] Wang I.J., Lin C.C., Lin Y.J., Hsieh W.S., Chen P.C. (2014). Early life phthalate exposure and atopic disorders in children: A prospective birth cohort study. Environ. Int..

[3] Wang I.J., Karmaus W.J., Chen S.L., Holloway J.W., Ewart S. (2015). Effects of phthalate exposure on asthma may be mediated through alterations in DNA methylation. Clin. Epigenetics.

[4] Wang I.J., Karmaus W.J. (2017). Oxidative Stress-Related Genetic Variants May Modify Associations of Phthalate Exposures with Asthma. Int. J. Environ. Res. Public. Health.

[5] Bølling A.K., Sripada K., Becher R., Bekö G. (2020). Phthalate exposure and allergic diseases: Review of epidemiological and experimental evidence. Environ. Int..

[6] Nuzzi G., Di Cicco M.E., Peroni D.G. (2021). Breastfeeding and Allergic Diseases: What’s New?. Children.

[7] Wang B., Liu F., Dong J., You M., Fu Y., Li C., Lu Y., Chen J. (2018). Maternal exposure to environmental DEHP exacerbated OVA-induced asthmatic responses in rat offspring. Sci. Total Environ..

[8] Lopez-Santamarina A., Gonzalez E.G., Lamas A., Mondragon A.D.C., Regal P., Miranda J.M. (2021). Probiotics as a Possible Strategy for the Prevention and Treatment of Allergies. A Narrative Review. Foods.

[9] Spacova I., Van Beeck W., Seys S., Devos F., Vanoirbeek J., Vanderleyden J., Ceuppens J., Petrova M., Lebeer S. (2020). *Lactobacillus rhamnosus* probiotic prevents airway function deterioration and promotes gut microbiome resilience in a murine asthma model. Gut Microbes.

[10] Lan H., Gui Z., Zeng Z., Li D., Qian B., Qin L.Y., Dai L., Song J.L. (2022). Oral administration of *Lactobacillus plantarum* CQPC11 attenuated the airway inflammation in an ovalbumin (OVA)-induced Balb/c mouse model of asthma. J. Food Biochem..

[11] Mennini M., Dahdah L., Artesani M.C., Fiocchi A., Martelli A. (2017). Probiotics in Asthma and Allergy Prevention. Front. Pediatr..

[12] Yang Y., Song X., Wang G., Xia Y., Xiong Z., Ai L. (2024). Understanding *Ligilactobacillus salivarius* from Probiotic Properties to Omics Technology: A Review. Foods.

[13] Messaoudi S., Manai M., Kergourlay G., Prévost H., Connil N., Chobert J.M., Dousset X. (2013). *Lactobacillus salivarius*: Bacteriocin and probiotic activity. Food Microbiol..

[14] Drago L., De Vecchi E., Gabrieli A., De Grandi R., Toscano M. (2015). Immunomodulatory Effects of *Lactobacillus salivarius* LS01 and *Bifidobacterium breve* BR03, Alone and in Combination, on Peripheral Blood Mononuclear Cells of Allergic Asthmatics. Allergy Asthma Immunol. Res..

[15] Dec M., Stępień-Pyśniak D., Puchalski A., Hauschild T., Pietras-Ożga D., Ignaciuk S., Urban-Chmiel R. (2021). Biodiversity of *Ligilactobacillus salivarius* Strains from Poultry and Domestic Pigeons. Animals.

[16] Neville B.A., O’Toole P.W. (2010). Probiotic properties of *Lactobacillus salivarius* and closely related Lactobacillus species. Future Microbiol..

[17] Krupa-Kotara K., Gwioździk W., Nandzik S., Grajek M. (2023). The Role of Microbiota Pattern in Anxiety and Stress Disorders—A Review of the State of Knowledge. Psych.

[18] Sohail M.U., Hedin L., Al-Asmakh M. (2021). Dysbiosis of the Salivary Microbiome is Associated with Hypertension and Correlated with Metabolic Syndrome Biomarkers. Diabetes Metab. Syndr. Obes..

[19] Jahreis S., Trump S., Bauer M., Bauer T., Thürmann L., Feltens R., Wang Q., Gu L., Grützmann K., Röder S. (2018). Maternal phthalate exposure promotes allergic airway inflammation over 2 generations through epigenetic modifications. J. Allergy Clin. Immunol..

[20] Lee M.C., Hsu Y.J., Ho H.H., Hsieh S.H., Kuo Y.W., Sung H.C., Huang C.C. (2020). *Lactobacillus salivarius* Subspecies salicinius SA-03 is a New Probiotic Capable of Enhancing Exercise Performance and Decreasing Fatigue. Microorganisms.

[21] Paveljšek D., Juvan P., Košir R., Rozman D., Hacin B., Ivičak-Kocjan K., Rogelj I. (2018). *Lactobacillus fermentum* L930BB and *Bifidobacterium animalis* subsp. animalis IM386 initiate signalling pathways involved in intestinal epithelial barrier protection. Benef. Microbes.

[22] Wittke A., Weaver V., Mahon B.D., August A., Cantorna M.T. (2004). Vitamin D receptor-deficient mice fail to develop experimental allergic asthma. J. Immun..

[23] Li C.Y., Lin H.C., Hsueh K.C., Wu S.F., Fang S.H. (2010). Oral administration of *Lactobacillus salivarius* inhibits the allergic airway response in mice. Can. J. Microbiol..

[24] Kay A.B., Ying S., Durham S.R. (1995). Phenotype of cells positive for interleukin-4 and interleukin-5 mRNA in allergic tissue reactions. Int. Arch. Allergy Immunol..

[25] Borish L., Aarons A., Rumbyrt J., Cvietusa P., Negri J., Wenzel S. (1996). Interleukin-10 regulation in normal subjects and patients with asthma. J. Allergy Clin. Immunol..

[26] Hawrylowicz C.M., O’Garra A. (2005). Potential role of interleukin-10-secreting regulatory T cells in allergy and asthma. Nat. Rev. Immunol..

[27] Greenfeder S., Umland S.P., Cuss F.M., Chapman R.W., Egan R.W. (2001). Th2 cytokines and asthma. The role of interleukin-5 in allergic eosinophilic disease. Respir. Res..

[28] Assa'ad A.H., Gupta S.K., Collins M.H., Thomson M., Heath A.T., Smith D.A., Perschy T.L., Jurgensen C.H., Ortega H.G., Aceves S.S. (2011). An antibody against IL-5 reduces numbers of esophageal intraepithelial eosinophils in children with eosinophilic esophagitis. Gastroenterology.

[29] Eum S.Y., Hailé S., Lefort J., Huerre M., Vargaftig B.B. (1995). Eosinophil recruitment into the respiratory epithelium following antigenic challenge in hyper-IgE mice is accompanied by interleukin 5-dependent bronchial hyperresponsiveness. Proc. Natl. Acad. Sci. USA.

[30] Tesciuba A.G., Subudhi S., Rother R.P., Faas S.J., Frantz A.M., Elliot D., Weinstock J., Matis L.A., Bluestone J.A., Sperling A.I. (2001). Inducible costimulator regulates Th2-mediated inflammation, but not Th2 differentiation, in a model of allergic airway disease. J. Immunol..

[31] Redmond W.L., Ruby C.E., Weinberg A.D. (2009). The role of OX40-mediated co-stimulation in T-cell activation and survival. Crit. Rev. Immunol..

[32] Mäkelä M.J., Kanehiro A., Borish L., Dakhama A., Loader J., Joetham A., Xing Z., Jordana M., Larsen G.L., Gelfand E.W. (2000). IL-10 is necessary for the expression of airway hyperresponsiveness but not pulmonary inflammation after allergic sensitization. Proc. Natl. Acad. Sci. USA.

[33] Kips J.C. (2001). Cytokines in asthma. Eur. Respir. J. Suppl..

[34] Bickert T., Trujillo-Vargas C.M., Duechs M., Wohlleben G., Polte T., Hansen G., Oelschlaeger T.A., Erb K.J. (2009). Probiotic *Escherichia coli* Nissle 1917 suppresses allergen-induced Th2 responses in the airways. Int. Arch. Allergy Immunol..

[35] Rodríguez-Nogales A., Algieri F., Garrido-Mesa J., Vezza T., Utrilla M.P., Chueca N., Fernández-Caballero J.A., García F., Rodríguez-Cabezas M.E., Gálvez J. (2018). The Administration of *Escherichia coli* Nissle 1917 Ameliorates Development of DSS-Induced Colitis in Mice. Front. Pharmacol..

[36] Yun X., Shang Y., Li M. (2015). Effect of *Lactobacillus salivarius* on Th1/Th2 cytokines and the number of spleen CD4⁺ CD25⁺ Foxp3⁺ Treg in asthma Balb/c mouse. Int. J. Clin. Exp. Pathol..

[37] Thorsen J., Rasmussen M.A., Waage J., Mortensen M., Brejnrod A., Bønnelykke K., Chawes B.L., Brix S., Sørensen S.J., Stokholm J. (2019). Infant airway microbiota and topical immune perturbations in the origins of childhood asthma. Nat. Commun..

[38] Teo S.M., Tang H.H.F., Mok D., Judd L.M., Watts S.C., Pham K., Holt B.J., Kusel M., Serralha M., Troy N. (2018). Airway Microbiota Dynamics Uncover a Critical Window for Interplay of Pathogenic Bacteria and Allergy in Childhood Respiratory Disease. Cell Host Microbe.

[39] Chiu C.J., Huang M.T. (2021). Asthma in the Precision Medicine Era: Biologics and Probiotics. Int. J. Mol. Sci..

[40] Zubeldia-Varela E., Barker-Tejeda T.C., Obeso D., Villaseñor A., Barber D., Pérez-Gordo M. (2022). Microbiome and Allergy: New Insights and Perspectives. J. Investig. Allergol. Clin. Immunol..

[41] Arrieta M.C., Stiemsma L.T., Dimitriu P.A., Thorson L., Russell S., Yurist-Doutsch S., Kuzeljevic B., Gold M.J., Britton H.M., Lefebvre D.L. (2015). Early infancy microbial and metabolic alterations affect risk of childhood asthma. Sci. Transl. Med..

[42] Abrahamsson T.R., Jakobsson H.E., Andersson A.F., Björkstén B., Engstrand L., Jenmalm M.C. (2014). Low gut microbiota diversity in early infancy precedes asthma at school age. Clin. Exp. Allergy.

[43] Hufnagl K., Pali-Schöll I., Roth-Walter F., Jensen-Jarolim E. (2020). Dysbiosis of the gut and lung microbiome has a role in asthma. Semin. Immunopathol..

[44] Kim Y.H., Park M.R., Kim S.Y., Kim M.Y., Kim K.W., Sohn M.H. (2023). Respiratory microbiome profiles are associated with distinct inflammatory phenotype and lung function in children with asthma. J. Investig. Allergol. Clin. Immunol..

